# Photo‐Rechargeable Organic Supercapacitor via Light‐Activated Electrolytes

**DOI:** 10.1002/advs.202500978

**Published:** 2025-04-25

**Authors:** Shubhra Kanti Bhaumik, Sudipta Biswas, Nitzan Shauloff, Ahiud Morag, Raz Jelinek

**Affiliations:** ^1^ Department of Chemistry Ben Gurion University of the Negev Beer Sheva 8410501 Israel; ^2^ Ilse Katz Institute for Nanotechnology Ben Gurion University of the Negev Beer Sheva 8410501 Israel; ^3^ Center for Advancing Electronics Dresden (CFAED) Faculty of Chemistry and Food Chemistry Technische Universität Dresden Mommsenstrasse 4 01069 Dresden Germany; ^4^ Department of Synthetic Materials and Functional Devices Max‐Planck Institute of Microstructure Physics 06120 Halle Germany

**Keywords:** organic supercapacitors, photoacids, photoactive electrolytes, photo‐rechargeable supercapacitors

## Abstract

Light‐mediated energy storage is key in diverse applications, including photonic devices, solar energy harvest, and others. Here, we demonstrate the construction of a photo‐rechargeable supercapacitor, in which light‐induced recharging is based, for the first time, on photoactive labile electrolytes. Specifically, the supercapacitor dielectric medium consisted of 2‐nitrobenzaldehyde as the electrolyte. In the dark, 2‐nitrobenzaldehyde is not ionized and the device displayed low capacitance. However, upon light irradiation, 2‐nitrobenzaldehyde undergoes chemical transformation and forms labile benzoic acid derivatives. These photoacids further ionize upon illumination, with the redox‐active photoinduced ionic species giving rise to significantly enhanced capacitance. Importantly, the generation of photoinduced electrolytes is reversible, facilitating multiple charge–discharge cycles. The photo‐rechargeable device exhibited extended discharge times, high specific capacitance, capacitance retention, and cyclic stability. The use of the photo‐rechargeable supercapacitor is demonstrated for practical charging and powering an external load. Light‐induced energy storage mediated by photoactive electrolytes is a new and powerful concept and may open new avenues for photo‐charged devices, solar energy harvesting, and storage.

## Graphical abstract

A photo‐rechargeable organic supercapacitor device is fabricated comprising activated charcoal electrodes and 2‐nitrobenzaldehyde as the electrolyte. The operation principle of the device relies on the photoinduced transformation of 2‐nitrobenzaldehyde into electrochemically active benzoic acid derivatives. The photogenerated ionic and redox‐active benzoic acid electrolytes furnish high ionic conductivity and specific capacitance, utilized for excellent performance of the supercapacitor device.

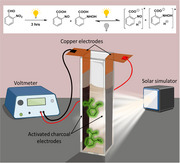

## 1 Introduction

Photo‐rechargeable energy storage modules constitute key components in diverse platforms and applications. Specifically, the intermittent nature of solar‐generated energy requires the integration of varied storage systems particularly batteries and supercapacitors (SCs).^[^ ^1^, ^2^, ^3^, ^4^ ^]^ Other examples of photo‐rechargeable energy storage devices include photo‐rechargeable hybrid supercapacitors, fuel cells, redox flow batteries (RFB), and Li‐ion batteries.^[^ ^5^, ^6^, ^7^ ^]^ The large majority of light‐induced energy storage systems constitute either externally linked solar cells and energy storage devices,^[^ ^8^, ^9^, ^10^, ^11^ ^]^ or the design of photo‐sensitive electrodes in which the electrochemical properties are enhanced upon light irradiation.^[^ ^12^, ^13^ ^]^ These strategies have inherent limitations and significant challenges. Energy storage devices such as batteries connected to photovoltaic cells, for example, display energy losses due to ohmic transport, resistive loss, low cyclic stability, suboptimal capacity, and complex fabrication methods.^[^ ^8^, ^14^, ^15^ ^]^

To address the challenges inherent in coupling solar cell modules with energy storage components, recent efforts have been aimed at developing single devices that integrate both light harvesting and energy storage. In supercapacitors, such devices have primarily focused on the design of photoactive electrodes exhibiting enhanced capacitive properties upon light illumination.^[^ ^16^ ^]^ Representative examples of such strategies include V_2_O_5_‐activated carbon electrode‐based photo‐rechargeable Zn‐ion capacitors,^[^ ^17^ ^]^ ZnO‐nanoflakes/rGO‐based solar‐assisted supercapacitors,^[^ ^18^ ^]^ photo‐assisted asymmetric supercapacitors based on ZnCo_2_O_4_ or CuCo_2_S_4_,^[^ ^19^ ^]^ and others. Such systems exhibit conceptual and practical challenges, including the extent of light absorption, inefficient photo‐response, inadequate electrochemical performance, and low stability.^[^ ^20^, ^21^ ^]^

Here, we demonstrate a new concept for photo‐rechargeable supercapacitors, in which, for the first time, a photoinduced transformation of the electrolyte, rather than the electrodes, constitutes the operation mechanism for light‐mediated energy storage. Specifically, the electrolyte in the device consists of a small molecule – 2‐nitrobenzaldehyde – which chemically transforms upon light irradiation into photoacid species. Photoacids exhibit enhanced dissociation in the photo‐excited states compared to their ground states.^[^ ^22^, ^23^, ^24^, ^25^ ^]^ We exploit this property to achieve significantly higher capacitance upon light irradiation (almost 100% increase), ascribed to the generation of photoinduced transient redox‐active ionic species. The photo‐rechargeable supercapacitor displays high specific capacitance (320 Fg^−1^ cm^−2^ in a 3‐electrode configuration, ≈90 Fg^−1^ cm^−2^ for a two‐electrode device), good cycling stability, and comparatively higher energy density upon light illumination. This innovative photo‐rechargeable supercapacitor design may open new avenues for light‐mediated energy storage in diverse applications.

## 2 Results and Discussion

**Figure 1:**
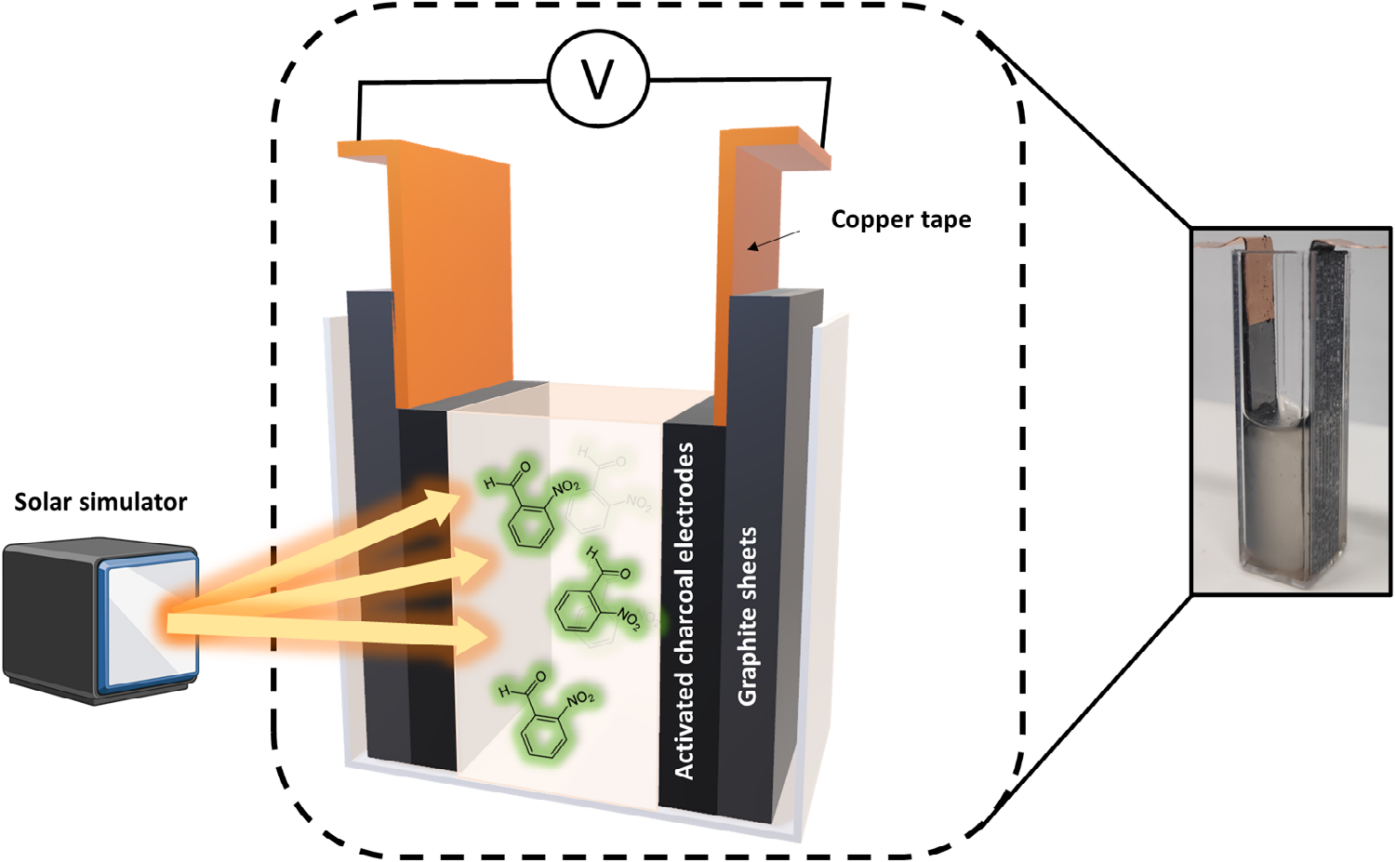
The photo‐rechargeable supercapacitor design. Activated charcoal electrodes were fitted on the sides of a transparent cuvette. The aqueous electrolyte consisted of the photolabile molecule 2‐nitrobenzaldehyde.

**Figure** **1** illustrates the photo‐rechargeable supercapacitor design. The two electrodes, consisting of activated charcoal deposited on graphite sheets, were placed at a 1 cm distance in a commercially available transparent plastic cuvette. The key photo‐activated component of the device was the electrolyte, comprising 2‐nitrobenzaldehyde, a photoacid known to undergo a chemical transformation upon light irradiation,^[^ ^26^, ^27^, ^28^ ^]^ dissolved in a water/DMSO/acetonitrile (4:2:1) solvent mixture.

**Figure 2:**
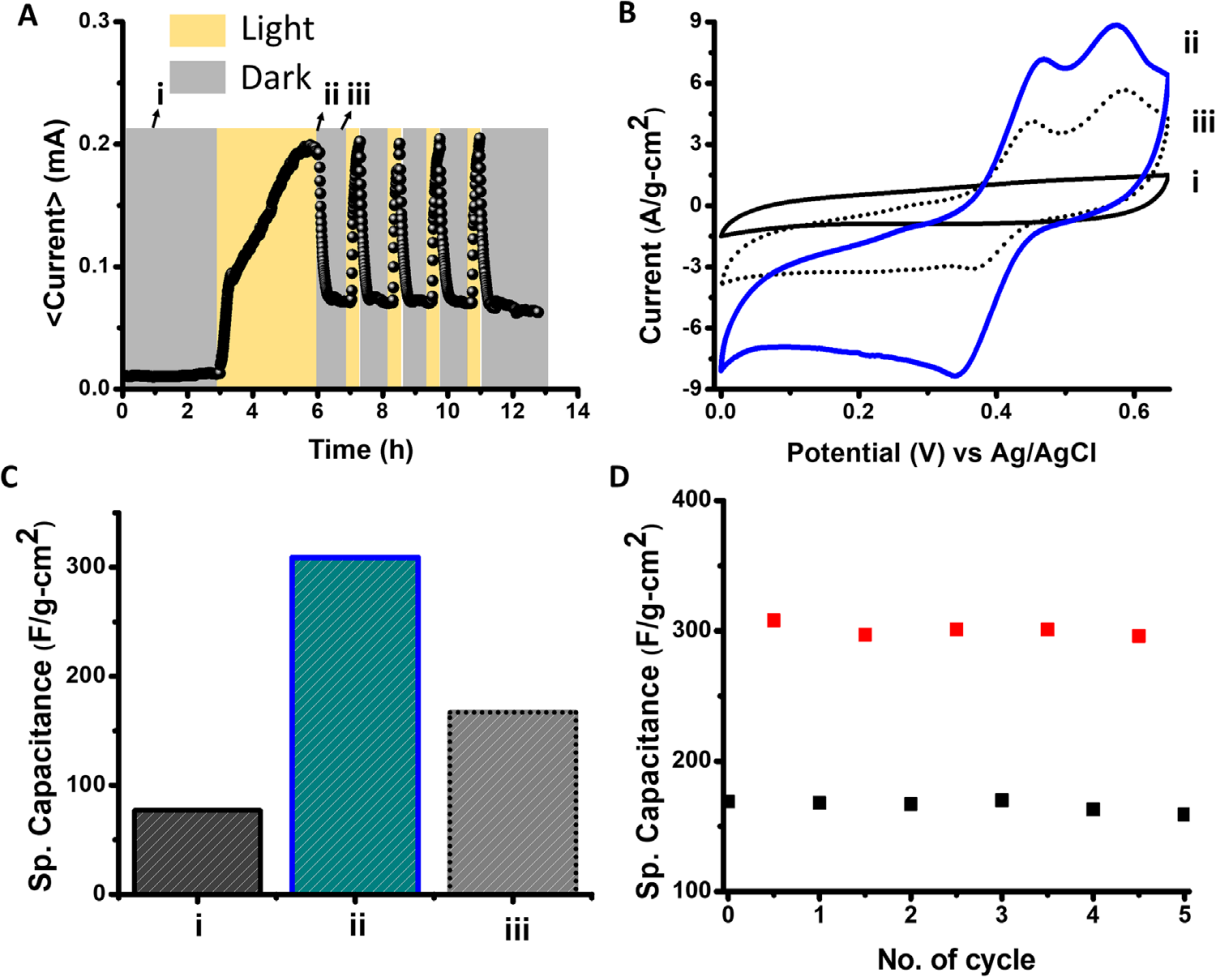
Light‐induced electrochemical properties of the 2‐nitrobenzaldehyde‐based supercapacitor. A) Photocurrent response in an aqueous solution comprising 0.6 m 2‐nitrobenzaldehyde: (i) upon initial dissolution of 2‐nitrobenzaldehyde, before light irradiation; (ii) 3 hours irradiation (WLED, 70 mW cm^−2^); (iii) illumination was turned off. B) CV curves recorded in a 3‐electrode setup at the different illumination states, scan rate of 10 mV s^−1^ (solution of 0.6 m 2‐nitrobenzaldehyde and 0.5 m H_2_SO_4_). C) Bar diagram comparing the specific capacitance calculated from the CV curves. D) Specific capacitance values in the dark (state iii, black squares) and upon light illumination (state ii; red squares) recorded in several consecutive cycles.

The electrochemical properties of the 2‐nitrobenzaldehyde‐based supercapacitor construct are depicted in **Figure** **2**, particularly the effects of light irradiation. We first investigated the photocurrent generated upon illumination of the 2‐nitrobenzaldehyde electrolyte (Figure 2A). Prior to light irradiation, a very low current was measured (around 10 µA) reflecting the absence of charged electrolyte species in the aqueous solution (except water hydrolyzed ions; Figure 2A,i). Subsequent light irradiation, employing a white light emitting diode (WLED; power of 70 mW cm^−2^), gave rise to a gradual current increase up to 200 µA after 3‐hour irradiation (Figure 2A,ii), likely accounting for the formation of photoinduced acidic constituents in the solution.^[^ ^26^, ^27^, ^28^ ^]^ Turning off illumination resulted in a rapid decrease in current, reaching a plateau at around 70 µA (Figure 2A,iii). This current, which is significantly higher than the initially recorded current of 10 µA, indicates the formation of a stable photoinduced ionic species. A subsequent light turn‐on (using the same light source) yielded a rapid increase in the recorded current, to around 200 µA (Figure 2A), reverting to 70 µA after switching off the light (Figure 2A). Figure 2A demonstrates that cycling of the light on–light off photocurrents could be successfully carried out.

Figure 2B–D depict the photoinduced capacitance properties in a 3‐electrode system employing 2‐nitrobenzaldehyde (0.6 m) supplemented by 0.5 m H_2_SO_4_ as the electrolyte (H_2_SO_4_ was added to enhance electrolyte conductivity). Figure 2B presents the cyclic voltammetry (CV) curves recorded at the specific light irradiation steps (e.g., i–iii) at a scan rate of 10 mV s^−1^, and corresponding calculated specific capacitance values are shown in Figure 2C. Echoing the current data in Figure 2A–C reveals dramatic photoinduced capacitive transformations. The initial CV curve recorded upon external power application at 0–0.65 V potential window (designed to ascertain that electrolyte electro‐dissociation does not occur) prior to light irradiation (Figure 2B,i) indicates low specific capacitance (≈75 Fg^−1^ cm^−2^, Figure 2C,i). The low capacitance likely accounts for 2‐nitrobenzaldehyde being an ineffective electrolyte, which is also reflected in the absence of redox peaks (Figure 2B,i).

Following the initial 3‐hr illumination, the recorded CV curve indicates a significantly higher capacitance of ≈320 Fg^−1^ cm^−2^ (Figure 2B,ii,C,ii). Furthermore, the CV curve (Figure 2B,ii) displays prominent oxidation peaks at 0.46 and 0.57 V accounting for the photoinduced chemical transformation of 2‐nitrobenzaldehyde into electrochemically active species. This result echoes the photocurrent data (i.e., Figure 2A,ii), indicating the generation of electrochemically active electrolytes. Subsequent turning off light irradiation resulted in a lower capacitance ≈170 Fg^−1^ cm^−2^ (Figure 2C,iii and 2B,iii). Similar light‐induced changes were also observed when a glassy carbon electrode was employed as the counter electrode, instead of a Pt wire (Figure S1, Supporting Information). The CV profiles and corresponding specific capacitance values at different scan rates are presented in Figures S2 and S3 and Table S1, Supporting Information. The specific capacitance of the 2‐nitrobenzaldehyde supercapacitor could be cycled between the dark/light conditions (Figure 2D), underscoring the reversibility and stability of the system. Importantly, no photo‐charging was observed in a comparable 3‐electrode system that consisted of only H_2_SO_4_ in the electrolyte solution (i.e., no 2‐nitrobenzaldehyde present; Figure S4, Supporting Information). Notably, heating the system did not yield enhanced capacitance, indicating no contribution of thermal phenomena (Figure S5, Supporting Information).

**Figure 3:**
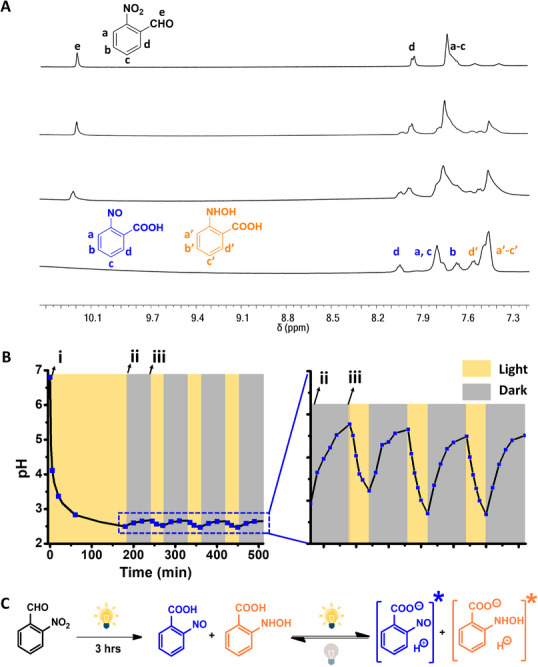
Photoinduced chemical transformations of the 2‐nitrobenzaldehyde electrolyte. A) ^1^H NMR spectra of 2‐nitrobenzaldehyde (1 mm) under dark (top spectrum) followed by WLED irradiation at different times (0–30 min). The assignment of respective peaks is indicated. B) pH of the aqueous 2‐nitrobenzaldehyde solution (0.6 m) under dark and light conditions. (i) Initial dissolution of 2‐nitrobenzaldehyde, before light irradiation; (ii) 3‐hour irradiation (WLED, 70 mW cm^−2^); (iii) light illumination turned off. C) Proposed photoinduced chemical transformation of 2‐nitrobenzaldehyde. Transient photoinduced ionized species are indicated by the stars.

The experiments presented in **Figure** **3** shed light on the mechanistic aspects of the photo‐induced capacitance enhancement. The time‐dependent ^1^H nuclear magnetic resonance (NMR) spectroscopy data in Figure 3A underscore the light‐induced chemical changes occurring in the 2‐nitrobenzaldehyde electrolyte solution. Specifically, the ^1^H NMR spectra in Figure 3A indicate that, upon WLED irradiation, 2‐nitrobenzaldehyde transformed into two molecular constituents, 2‐nitrosobenzoic acid and 2‐(hydroxyamino)benzoic acid. This chemical transformation is further corroborated by UV–vis^[^ ^29^ ^]^ and FTIR experiments (Figure S6, Supporting Information). This chemical transformation also manifests in the in situ micro‐pH measurements in light off / light on conditions (Figure 3B). Specifically, following the initial 3‐hour illumination step, we observe a significant drop in pH, from an almost neutral solution accounting for soluble 2‐nitrobenzaldehyde to a pH of 2.5 after light irradiation, accounting for the photoinduced benzoic acid species (e.g., Figure 3A). The photoinduced formation of ionizable 2‐nitrosobenzoic acid and 2‐(hydroxyamino)benzoic acid accounts for the enhanced current (Figure 2A) and capacitance (Figure 2B,C). Indeed, the redox peaks obtained in the cyclic voltammogram after irradiation (i.e., Figure 2B,ii,iii) most likely correspond to reactions of the nitroso‐ and hydroxyamino units, respectively, of the photoacids.^[^ ^30^, ^31^, ^32^ ^]^

Importantly, Figure 3B reveals that, after the initial light irradiation step, further recyclable light‐on / light‐off associated pH changes were apparent. Specifically, reversible photoinduced pH decrease was recorded upon light irradiation of the 2‐nitrosobenzoic acid / 2‐(hydroxyamino)benzoic acid mixture. These photoinduced reversible pH changes likely account for the enhanced photocurrent and capacitance enhancement cycles (i.e., Figure 2A–C), as light absorbance by the benzoic acid derivatives gave rise to the generation of transient ionized species. Indeed, light‐induced ionization of aromatic photoacids has been reported.^[^ ^33^, ^34^, ^35^ ^]^

The overall reaction scheme in Figure 3C underlines the chemical basis for the remarkable photoinduced electrochemical properties outlined in Figure 2. Specifically, 2‐nitrobenzaldehyde is a neutral molecule that, as the electrolyte constituent, contributes minimally to charge transport (thus low current, Figure 2A,i) and redox reactions (low capacitance, Figure 2B,i). In the second step, light irradiation gives rise to the chemical transformation of 2‐nitrobenzaldehyde into the two benzoic acid species, 2‐nitrosobenzoic acid and 2‐(hydroxyamino)‐benzoic acid. Notably, the two photogenerated acids are partly ionized in the dark condition in the aqueous solution, generating a comparatively higher current (Figure 2A,iii) and enhanced capacitance (e.g., Figure 2B,iii). Lastly, enhanced capacitance under light (Figure 2B,ii), which was fully reversible (Figure 2D), was likely linked to transient ionic species generated through light‐induced ionization of the photoacids.

**Figure 4:**
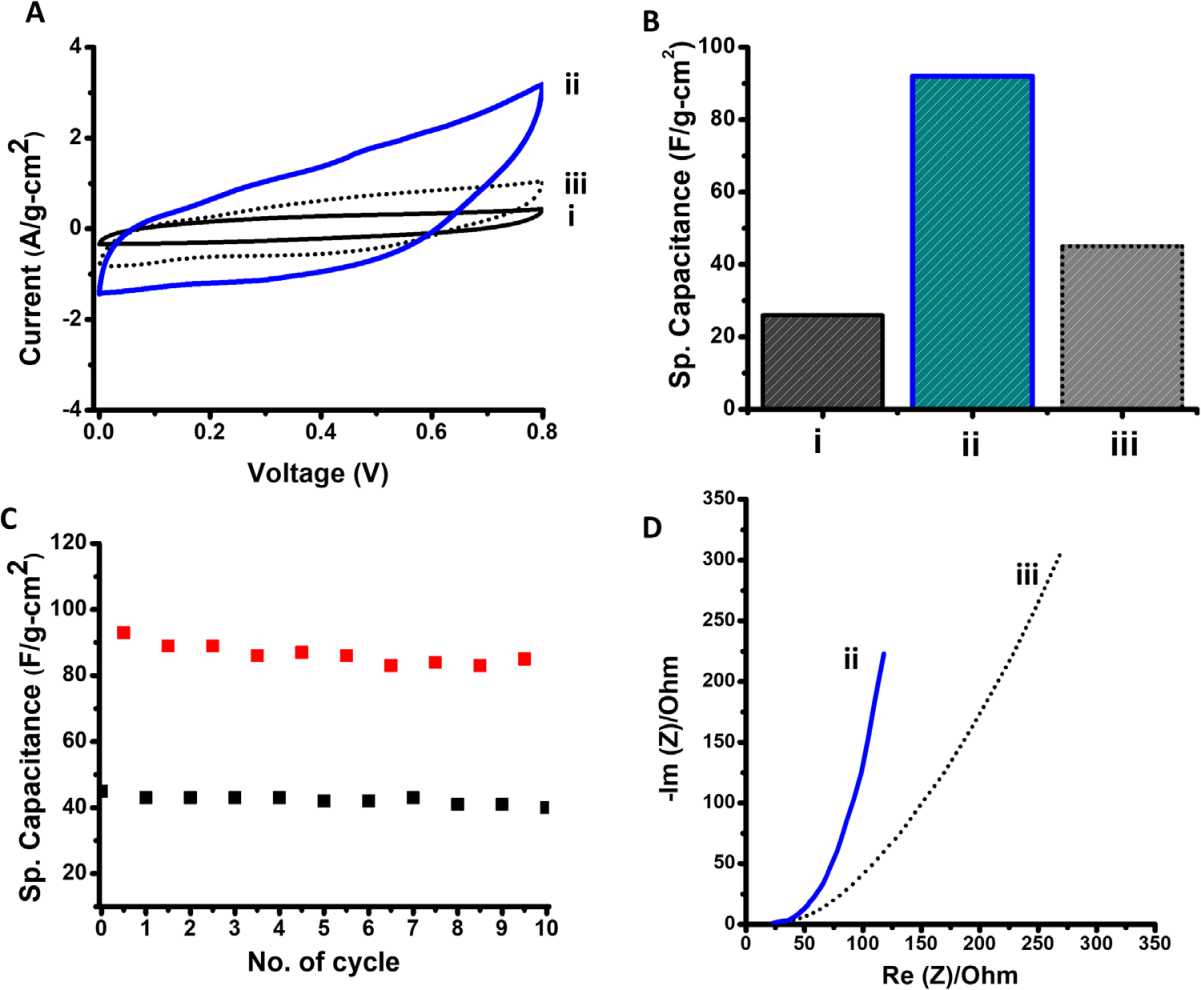
Photoinduced capacitance properties of a two‐electrode activated charcoal / 2‐nitrobenzaldehyde supercapacitor. A) CV curves of the device. (i) Initial dissolution of 2‐nitrobenzaldehyde, before light irradiation; (ii) 3 hours irradiation (WLED, 70 mW cm^−2^); (iii) illumination turned off. B) Bar diagram comparing the specific capacitance calculated from CV curves in (A). C) Specific capacitance values in the dark (black squares) and upon light illumination (red square) recorded in several cycles. D) Nyquist plots of states (ii) and (iii), recorded at a frequency range 50 mHz – 200 KHz.

**Figure 5:**
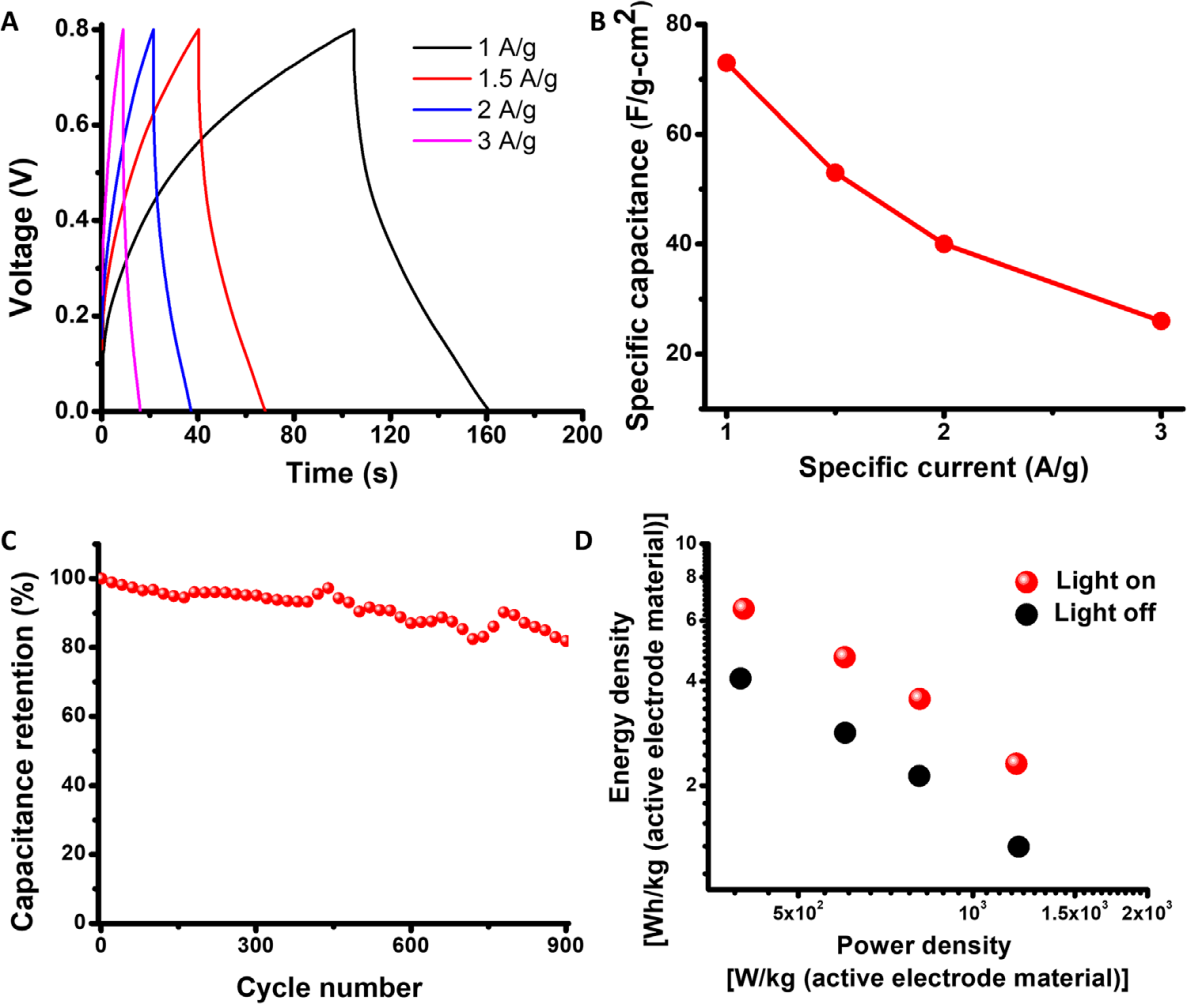
Electrochemical properties of light‐irradiated two‐electrode activated charcoal / 2‐nitrobenzaldehyde supercapacitor A) GCD curves recorded upon light irradiation in different current densities. B) Specific capacitance versus current density curves obtained from GCD curves. C) Cycling stability of the device (under illumination). D) Ragone plot of the device with and without light illumination.

**Figures** **4** and **5** depict the electrochemical properties of a photo‐rechargeable symmetric supercapacitor comprising two activated charcoal electrodes and 2‐nitrobenzaldehyde (supplanted with H_2_SO_4_) electrolyte in water/DMSO/acetonitrile (4:2:1) solvent mixture. Figure 4A presents CV curves recorded in different light‐off/light‐on steps (e.g., Figure 2A), at a scan rate of 10 mV s^−1^ in an optimized 0.8 V voltage window. The corresponding specific capacitance values calculated from the CV curves are outlined in Figure 4B. The nature of the CV curves suggested higher resistivity of the system due to the presence of organic electrolytes and organic solvents. The capacitance data in Figure 4A,B echo the results of the 3‐electrode system (e.g., Figure 2A,B), demonstrating a significant photoinduced capacitance enhancement. Specifically, the specific capacitance of the device prior to the initial photoactivation step was 25 Fg^−1^ cm^−2^ (Figure 4A,i,B,i), increasing to 92 Fg^−1^ cm^−2^ following 3‐hour illumination (Figure 4A,ii,B,ii), reverting to 45 Fg^−1^ cm^−2^ upon turning off light illumination (Figure 4A,iii,B,iii). CV experiments at different scan rates are presented in Figures S7 and S8 and Table S2 in Supporting Information. Light on / off cycling experiments in Figure 4C illustrate both the pronounced photoinduced capacitance enhancement effect, as well as the reversibility and stability of the system. Similar photoinduced capacitance changes were also manifested in a device prepared using quartz cuvette, instead of plastic cuvette (Figure S9, Supporting Information). In addition, negligible thermal effects were apparent for the 2‐electrode device (Figure S10, Supporting Information).

Figure 4D presents electrochemical impedance spectroscopy (EIS) measurements, carried out after initial photoactivation, at light‐on (ii) and light‐off conditions (iii), at a frequency range of 50 mHz–200 kHz with a sinus voltage amplitude of 10 mV. Upon fitting to a non‐ideal capacitor (the scheme of the electrical circuit is shown in Figure S11, Supporting Information), the calculated equivalent series resistance (*R*_ESR_) and charge transfer resistance (*R*_CT_) were 21.7 and 6.9 Ω, respectively, upon 3 hrs light illumination (ii), which are significantly lower than the respective resistance values in the dark (iii), i.e., 32.9 Ω and 13.9 Ω. The photoinduced decrease in solution resistance is consistent with the greater ionic conductivity (i.e., Figure 2A), while the reduction in R_CT_ under light illumination indicates more efficient redox reactivity of the ionized photoacids at the electrode surface.^[^ ^36^, ^37^ ^]^ The overall decrease in the resistance values contributes to the enhanced capacitance (Figure 2B,C), accounting for the superior electrochemical performance of the device upon light irradiation.

Galvanostatic charge‐discharge (GCD) analysis in Figure 5 furnishes further electrochemical characterization of the device and photo‐charging properties. The GCD curves recorded in the range of 1–3 A g^−1^ within a voltage window of 0.8 V for the device under illumination are depicted in Figure 5A. The GCD curves displayed relatively insignificant redox shoulders, a common feature in two‐electrode supercapacitor devices. Figure 5A also indicates pronounced discharge times at lower specific currents, under illumination. For example, at 1 A g^−1^ the discharge time was ≈60 sec, underscoring the specific capacitance of 73 Fg^−1^ cm^−2^ (Figure 5B). Figure S12 and Table S3 in Supporting Information, demonstrate that the discharge times were significantly higher upon light irradiation as compared to dark conditions. Figure 5B further depicts the specific capacitance at different specific currents, calculated from the GCD curves. The graph in Figure 5B shows a specific capacitance of 26 Fg^−1^ cm^−2^ at a higher specific current of 3 A g^−1^. The capacitance retention of more than 35% in triply higher current density attests to the feasible operation of the device in high power. The self‐discharge profile of the device (Figure S13, Supporting Information) displayed limited self‐discharge time probably due to higher resistance of the organic electrolyte system.

The cycling analysis in Figure 5C, recorded under light illumination, underscores good stability, displaying ≈85% capacitance retention after 900 cycles. The Ragone plot in Figure 5D indicates higher energy densities (at the same power densities) under light compared to dark conditions. The greater energy density obtained in the device under illumination signifies the effective applicability of the device as an energy source when irradiated.

**Figure 6:**
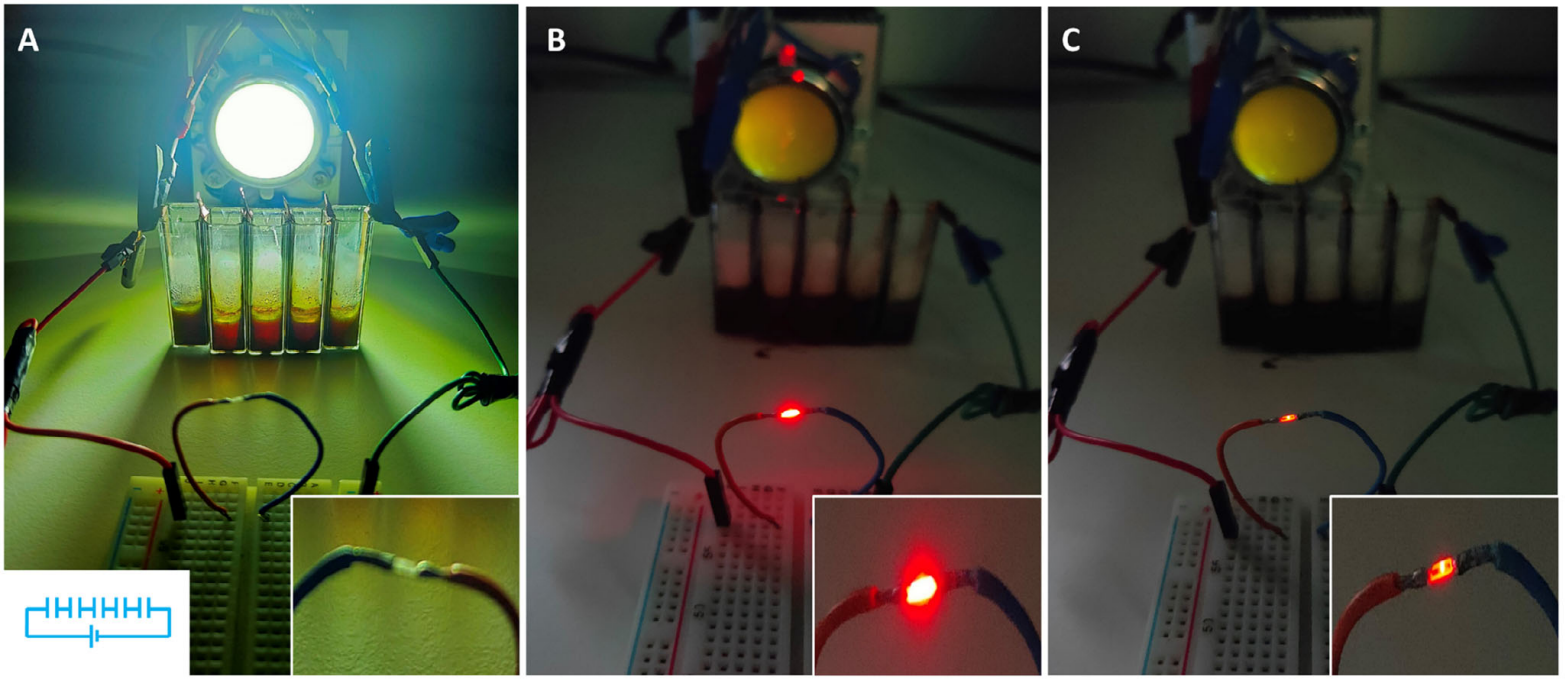
Utilizing the 2‐nitrobenzaldehyde supercapacitor for photoinduced charging and powering an external load. A) Photograph of charging/photoactivation of a device comprising five supercapacitors connected in series. The circuit diagram depicting five devices connected in series is shown on the bottom left, while the magnified LED is shown on the bottom right. B) Powering of the LED after turning off the light and disconnecting external charging. C) LED emission is still observed two minutes after disconnection.

**Figure** **6** depicts the practical utilization of the photoacid‐electrolyte supercapacitor. In the experiment, we linked five devices in series (diagram in the bottom left), designed to generate a sufficiently high voltage to turn on a light‐emitting device (LED). Following the initial 3‐hour photoactivation, we charged the device using a specific current of 1 A g^−1^, with concurrent white light illumination (light intensity of 70 mW cm^−2^) (Figure 6A). Subsequently, we ceased charging and connected a white LED to the supercapacitors (Figure 6B). Indeed, the photograph in Figure 6B shows that the LED was turned on upon connecting to the assembled devices. Notably, LED emission was retained for ≈2 min after turning off light irradiation (Figure 6C) manifesting the high energy density of the photo‐rechargeable supercapacitor.

Overall, the electrochemical experiments in Figures 3, 4, 5, 6 underscore a new concept for photo‐induced charging of a supercapacitor, in which significantly enhanced capacitance occurs, for the first time, through the photoinduced transformation of the electrolyte, rather than the electrodes. Indeed, we demonstrate that 2‐nitrobenzaldehyde – a non‐ionic, ineffective electrolyte – chemically transforms upon light irradiation into photoacid species. Consequently, we exploit the light‐induced dissociation of the photoacids to achieve significantly higher capacitance (almost 100% increase), ascribed to the generation of photoinduced transient redox‐active ionic species. The photo‐rechargeable supercapacitor displays high specific capacitance (320 Fg^−1^ cm^−2^ in a 3‐electrode configuration, ≈90 Fg^−1^ cm^−2^ for a two‐electrode device), good cycling stability, and comparatively higher energy density upon light illumination. Notably, this innovative strategy does not require transparency of the electrodes or the current collector. Moreover, this approach is environment‐friendly, cost‐effective, and does not require complex fabrication methods of light‐sensitive electrodes.

## 3 Conclusion

We report a photo‐rechargeable supercapacitor design, based, for the first time, on the generation of electrochemically active electrolytes upon light illumination. The organic supercapacitor employed an aqueous solution of 2‐nitrobenzaldehyde as the electrolyte and activated charcoal as electrodes. Notably, significantly enhanced capacitance was obtained upon photoinduced transformation of the non‐ionic 2‐nitrobenzaldehyde into labile benzoic acid derivatives, further yielding redox‐active light‐induced transient ionic species. The photo‐rechargeable device exhibited extended discharge times, high specific capacitance, good capacitance retention, and cyclic stability, making it amenable for practical energy storage and photo‐charging applications. The use of photoactive electrolytes as the key feature of light‐mediated energy storage devices is innovative, and may open new avenues for solar energy utilization, light charging of portable devices, and other applications.

## 4 Experimental Section

### Materials

All the reagents were purchased from external vendors and used as received. 2‐nitrobenzaldehyde was purchased from Aaron Chemicals LLC (China). Activated charcoal powder and poly(vinylidene fluoride) (*M*_w_ ≈ 534 000) were purchased from Sigma‐Aldrich (France). Graphite sheets (0.25 mm thickness) were purchased from Furnell (Ireland). Dimethyl sulfoxide (DMSO, 99.9% purity, analytical grade) was purchased from Fischer Chemicals (Switzerland). Acetonitrile (ACN, analytical grade) was purchased from Frutarom Ltd (Israel). Sulfuric acid (H_2_SO_4_, Conc.) was purchased from Merck. Doubly filtered water was used in the experiments using a Barnstead D7382 water purification system (Barnstead Thermolyne, USA), at a resistivity of 18.2 MΩ·cm.

### Instrumentation—Electrochemical Experiments

Cyclic voltammetry (CV) measurements were performed on a BioLogic SP‐150 instrument (Seyssinet‐Pariset, France), in a potential range of 0–0.65 V for 3 electrode measurements and 0–0.8 V for the 2‐electrode device. Galvanostatic charge‐discharge (GCD) measurements were carried out at current densities in the range of 1–3 A g^−1^ in a voltage window of 0.8 V for the device. Electrochemical impedance measurements were carried out between 50 mHz – 200 kHz with a sinus amplitude of 10 mV.

### Instrumentation—Photo‐Response Measurements

A white LED (WLED) was used as the light source (100 W, China). Light intensity was measured using a power meter (PM100, Thorlabs) while applying the illumination. To deliver similar power to a specific area, we used a focusing lens to control the beam size and shape, which can help ensure the selection of the irradiation area. The power level was adjusted by varying the input power using attenuators to reduce the power as needed. The light resource was set to 70 mW cm^−2^.

### Instrumentation—pH Measurements

Light‐induced pH changes were measured using a pH meter (CyberScan pH 510, Eutech Instruments, Thermo Scientific, Walthman, MA).

### Instrumentation—NMR Experiments

^1^H NMR was performed on a Bruker 500 spectrometer. Detailed ^1^H NMR values are described in the Supporting Information.

### Electrode Preparation

Strips of graphite sheets were first treated with 1 m HCl and 1 m Na_2_CO_3_ subsequently and then washed with doubly filtered water. This process was repeated thrice. The strips were subsequently dried at 100 °C overnight and the weights of the sheets were measured. Activated charcoal powder (47.5 mg) was added to a solution of poly(vinylidene fluoride) (2.5 mg) in acetone (15 mL) and stirred at 60 °C for 2 hr until a well‐mixed ink was formed. Then, the ink was drop‐casted on the dried graphite sheet strips and dried at 70 °C overnight to get the activated charcoal‐coated electrodes with a coating area of 1 cm^2^. The weights of the activated charcoal‐coated electrodes were measured and the weight of the coating materials in each strip was determined by subtracting the weight of the dried bare graphite sheets. The average weight of the coating material was 0.5 mg per electrode (1 cm^2^ area).

### 3‐Electrode Experiments

For 3‐electrode measurements, 3.5 mL transparent reusable plastic cuvettes of 1 cm path length, Ag/AgCl (3.5 m KCl) as reference electrode, Pt wire (0.5 cm diameter) as the counter electrode, and activated charcoal‐coated graphite sheet as working electrode were dipped into the electrolyte solution containing 2‐nitrobenzaldehyde (0.6 m) and H_2_SO_4_ (0.5 m) in water/DMSO/ACN (4:2:1) through a specially designed cap hole. The electrodes were externally connected with wires to a potentiometer.

### 2‐Electrode Device Fabrication

For the device fabrication, on the two opaque sides of a 3.5 mL transparent reusable plastic cuvette of 1 cm path length, two activated charcoal‐coated graphitic carbon electrodes were attached by carbon tapes. An electrolyte solution containing 2‐nitrobenzaldehyde (0.6 m) and H_2_SO_4_ (0.5 m) in water/DMSO/ACN (4:2:1) was placed inside it. Finally, two copper tapes were attached to the uncoated area of the graphite sheet and connected to a potentiometer.

### Electrochemical Data Analysis

Electrochemical parameters such as specific capacitance, energy density, and power density calculations are followed by established protocols.^[^ ^38^ ^]^ The equations used to calculate these values are described in the Supporting Information.

## Conflict of Interest

The authors declare no conflict of interest.

## Supporting information



Supporting Information

## Data Availability

The data that support the findings of this study are available from the corresponding author upon reasonable request.
