# Supplementary material for: Photo‐Rechargeable Organic Supercapacitor via Light‐Activated Electrolytes
Source: Adv Sci (Weinh). 2025 Apr 25;12(28):2500978. doi: 10.1002/advs.202500978 (PMC12302602; doi:10.1002/advs.202500978)
Supplement: Supplementary file 1 — Supporting Information [file ADVS-12-2500978-s001.docx]

Supporting Information

Photo-Rechargeable Organic Supercapacitor via Light-Activated Electrolytes

Shubhra Kanti Bhaumik, Sudipta Biswas, Nitzan Shauloff, Ahiud Morag, and Raz Jelinek*

Methods

***Electrochemical data analysis:*** Established protocols were followed to calculate the electrochemical parameters.^[1]^ The speciﬁc capacitance (C) values were calculated from the cyclic voltammetry (CV) curves recorded in three-electrode conﬁgurations and were estimated from Equation (1)

$C= \int\frac{\mathrm{IdV}}{2mvV}$ (1)

whereas the speciﬁc capacitance of the device is calculated from Equation (2)

$C= \int\frac{\mathrm{IdV}}{\mathrm{mvV}}$ (2)

The speciﬁc capacitance from the galvanostatic charge/discharge (GCD) curves from three electrodes, as well as the device, was calculated using Equation (3)

$C=\frac{I\triangle t}{\mathrm{mV}}$ (3)

The energy density (E) of the electrode was calculated using Equation (4)

$E=\frac{1}{2}CV^{2}$ (4)

The Power density (P) was calculated using Equation (5)

$P=\frac{E}{\triangle t}$ (5)

where I is the current at a speciﬁc potential, V is the potential/voltage window, m is the mass of the active electrode material, v is the scan rate at which CV is performed, and Δt is the discharge time obtained from the GCD.

Results and discussion

***^1^H NMR before light irradiation (500 MHz, D_2_O:DMSO-D_6_ = 4:3):*** δ (ppm)= 7.90-7.93 (m, 3H), 8.14 (d, *J* = 10 Hz, 1 H), 10.23 (s, 1H).

***^1^H NMR after light irradiation (500 MHz, D_2_O:DMSO-D_6_ = 4:3):*** δ (ppm)= 7.47-7.51 (m, 3H), 7.57 (d, *J* = 10 Hz, 1H) [corresponds to 2-(hydroxyamino)benzoic acid]; 7.68 (t, *J* = 8 Hz, 1H), 7.80-7.84 (m, 2H), 8.05 (d, *J* = 10 Hz, 1H) [corresponds to 2-nitrosobenzoic acid].


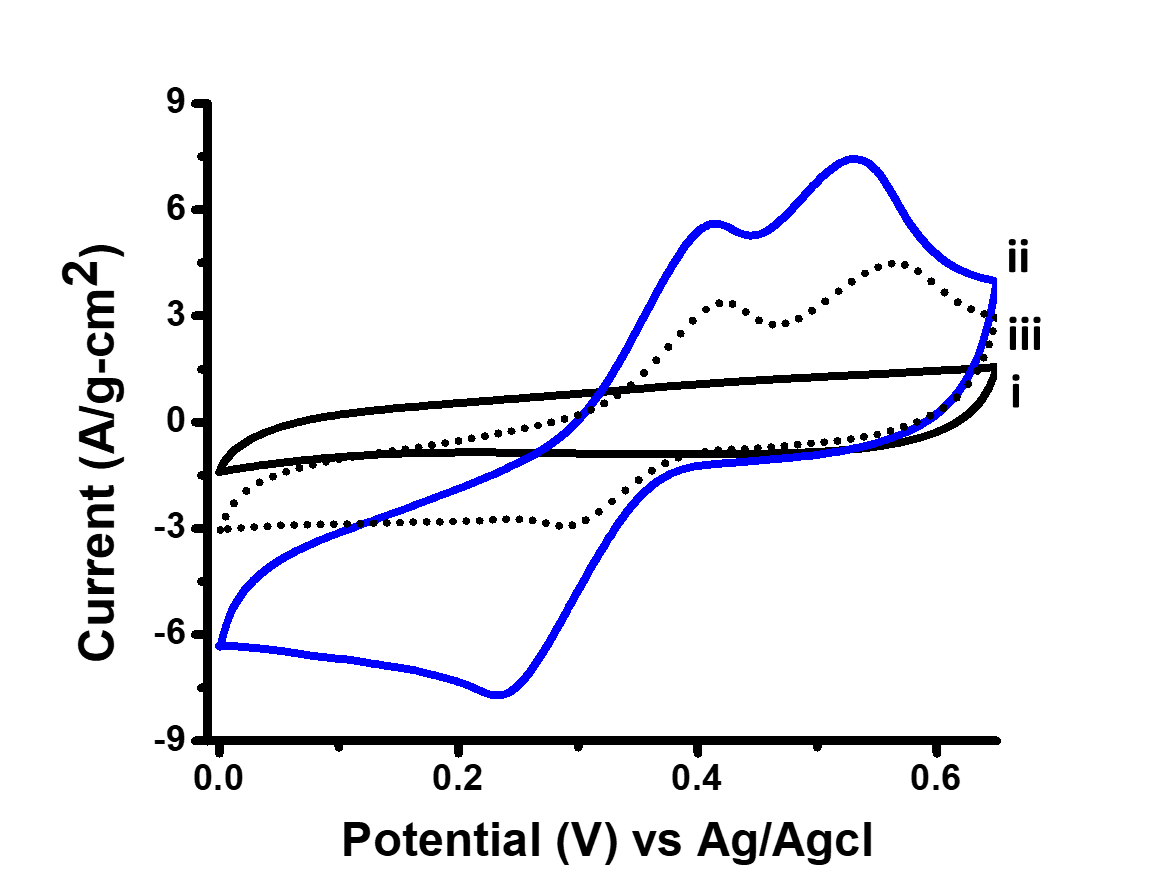


**Figure S1.** CV profiles of the system in a 3-electrode measurement for states **i**, **ii**, **iii** at 10 mV/s scan rate using activated charcoal as the working electrode, Ag/AgCl as the reference electrode, and glassy carbon as the counter electrode.

**
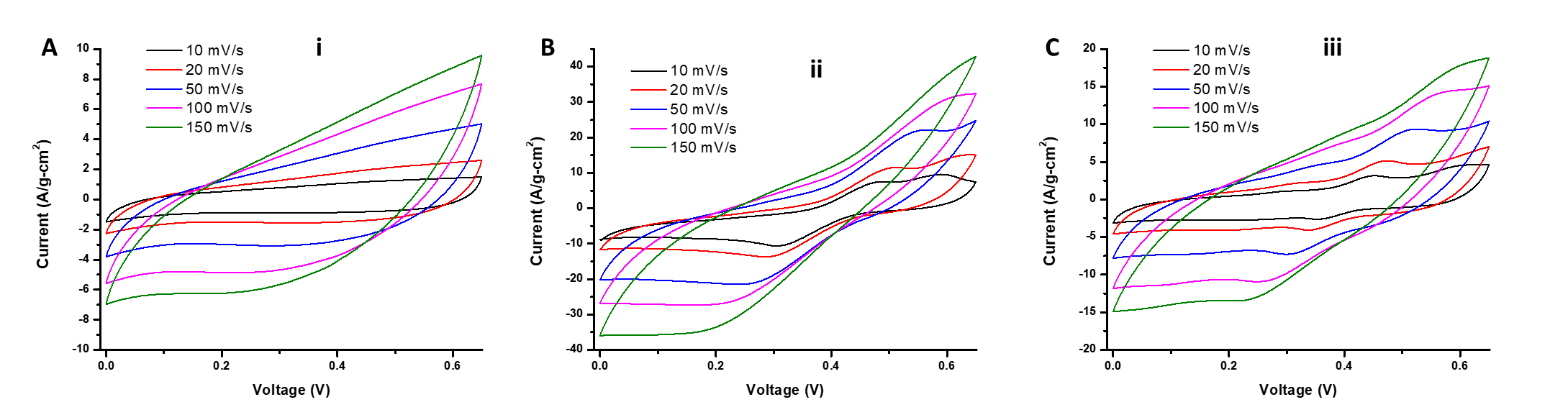
**
**Figure S2.** CV profiles of the system in a 3-electrode measurement for **A.** state **i**, **B.** state **ii**, and **C.** state **iii** at different scan rates.


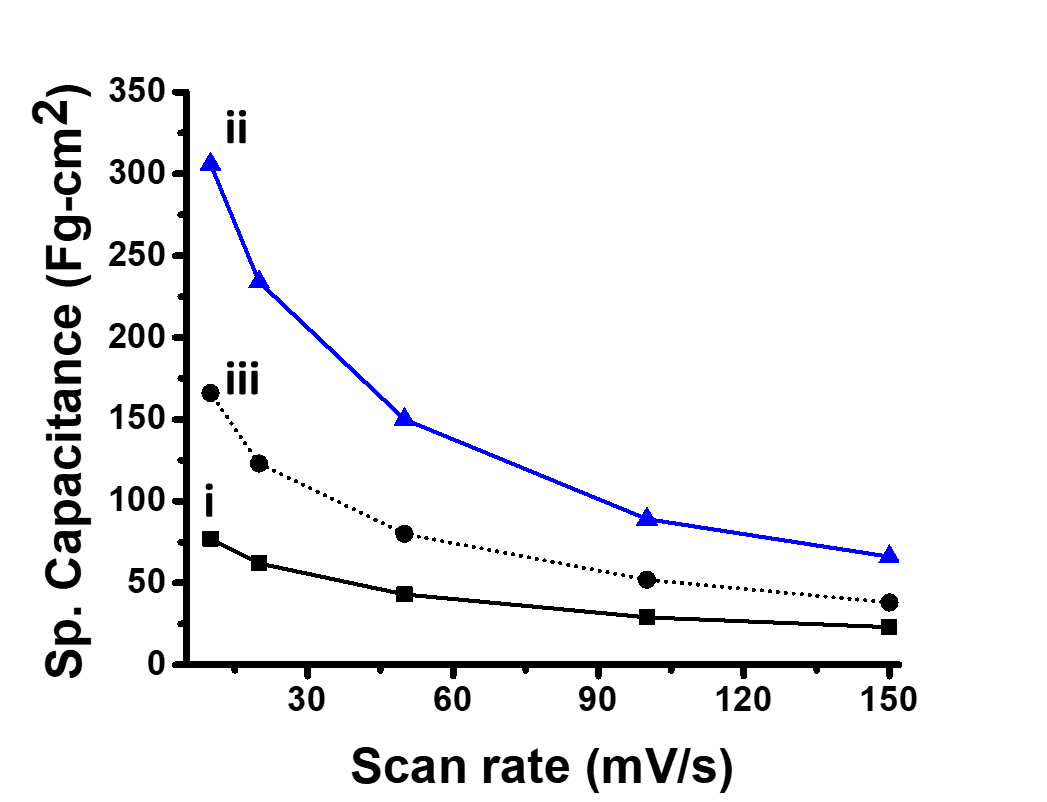


**Figure S3.** Specific capacitance vs scan rate plot for states **i**, **ii**, and **iii** in a 3-electrode measurement.

**Table S1.** Specific capacitance values at different scan rates for states **i**, **ii**, and **iii** in a 3-electrode measurement.

| Scan rate (mV/s) | Specific Capacitance for state i (Fg^-1^cm^-2^) | Specific Capacitance for state ii (Fg^-1^cm^-2^) | Specific Capacitance for state iii (Fg^-1^cm^-2^) |
| --- | --- | --- | --- |
| 10 | 75 | 320 | 170 |
| 20 | 62 | 234 | 123 |
| 50 | 43 | 150 | 80 |
| 100 | 29 | 89 | 52 |
| 150 | 23 | 66 | 38 |


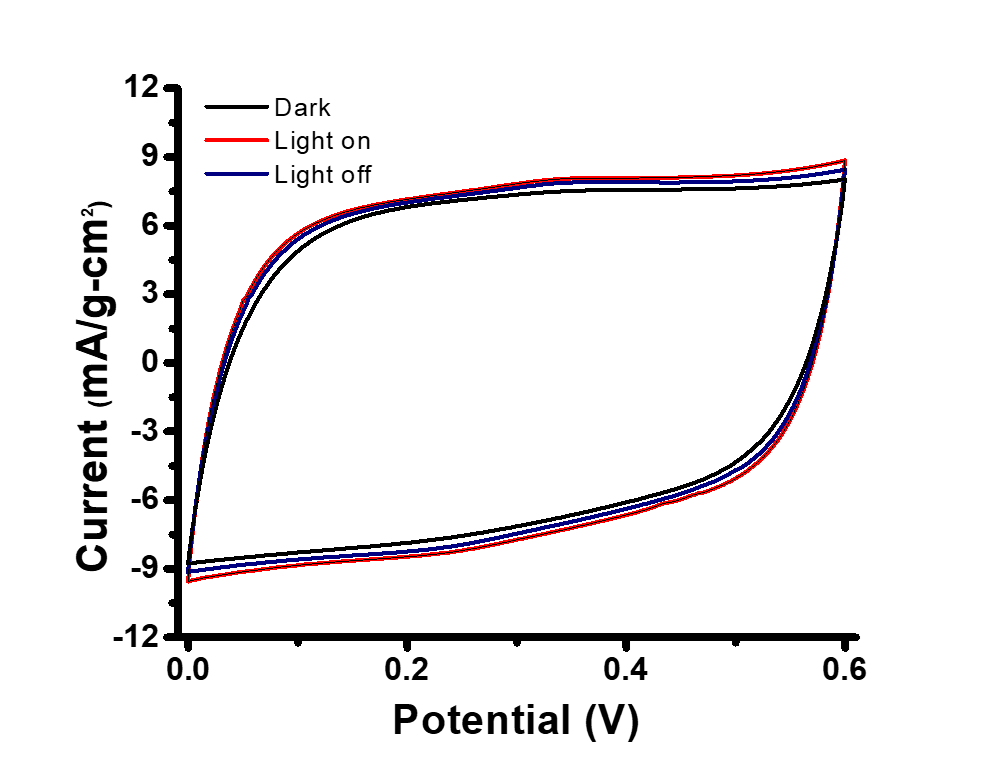


**Figure S4.** CV profile for a control system with 3-electrode measurement using activated charcoal electrode and 0.5 M H_2_SO_4_ in water/DMSO/ACN (4:2:1) under dark, light on and light off conditions.


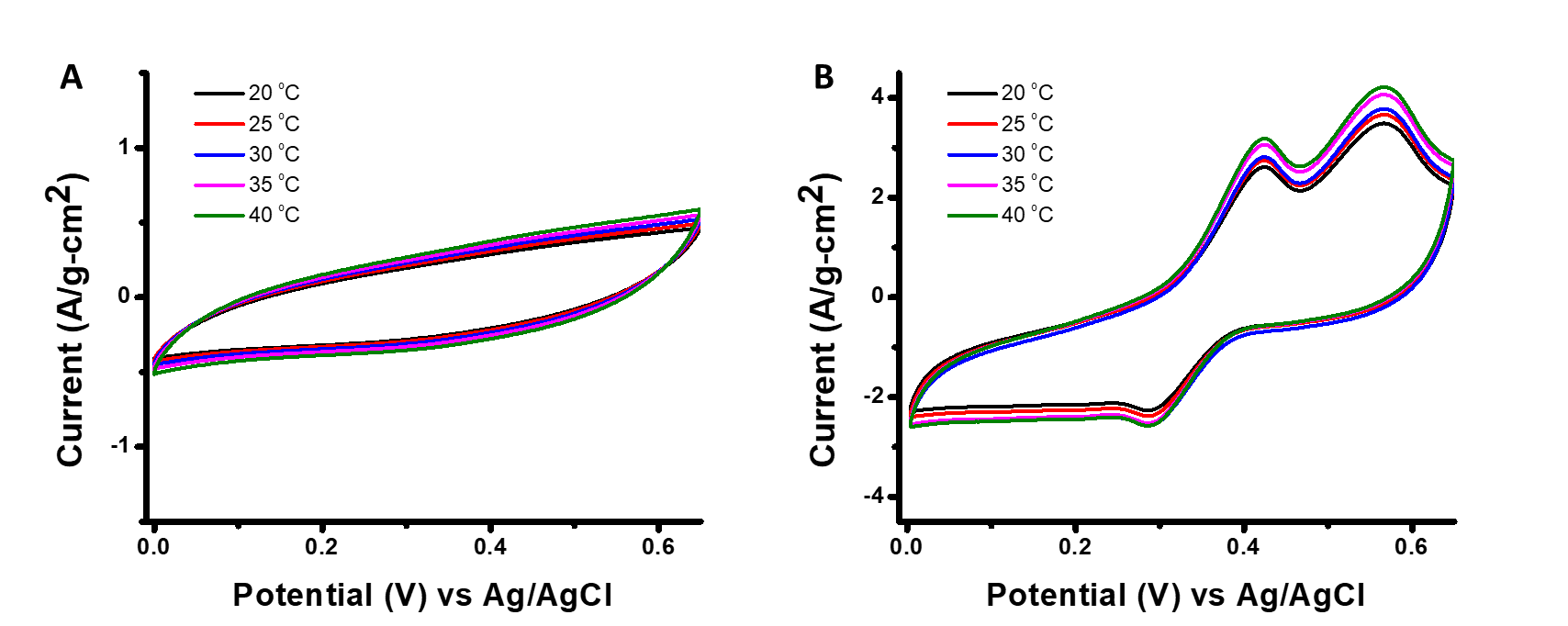


**Figure S5.** CV profiles for **A.** state **i** and **B.** state **iii** with 3-electrode measurement using an activated charcoal electrode and 0.5 M H_2_SO_4_ in water/DMSO/ACN (4:2:1) at different temperatures.


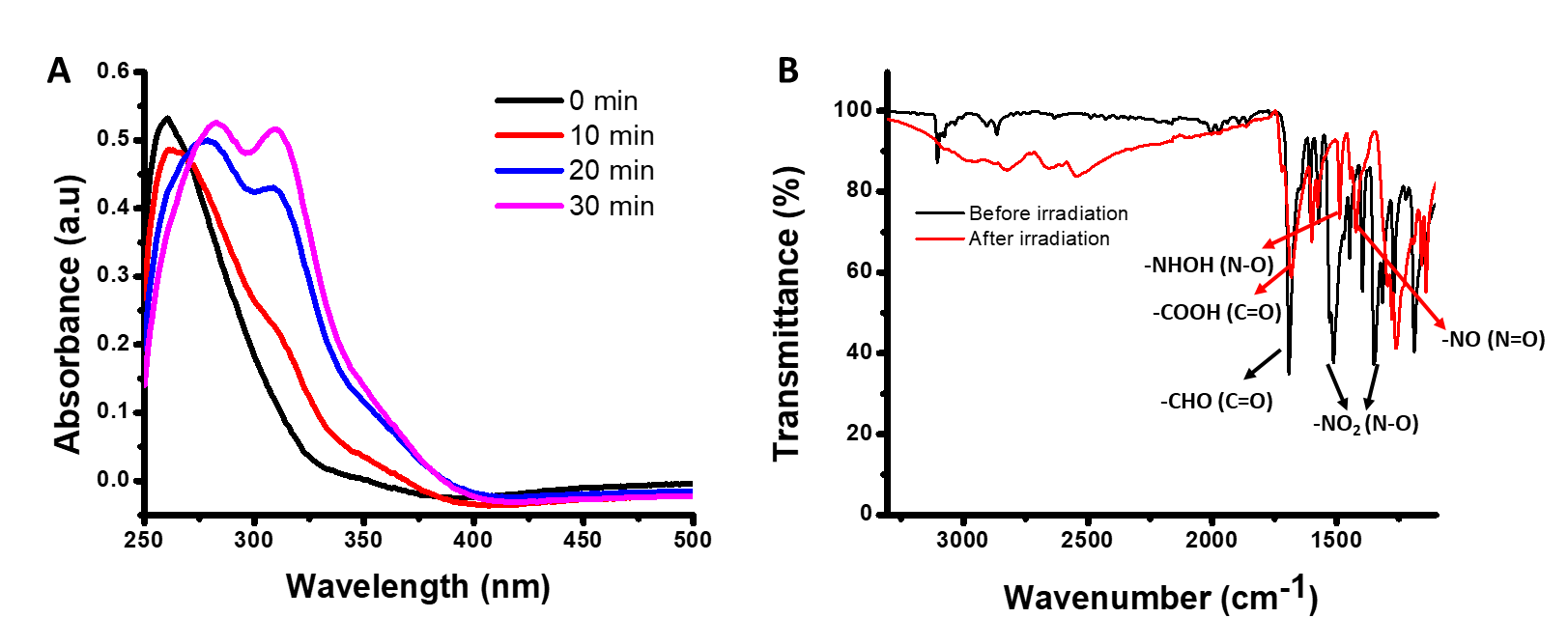


**Figure S6.** **A.** UV-vis spectral changes of 2-nitrobenzaldehyde diluted from different time light-irradiated samples (1 mM) in water/DMSO/ACN. **B.** ATR-IR spectra of the solid 2-nitrobenzaldehyde and the solid photo-irradiated products.


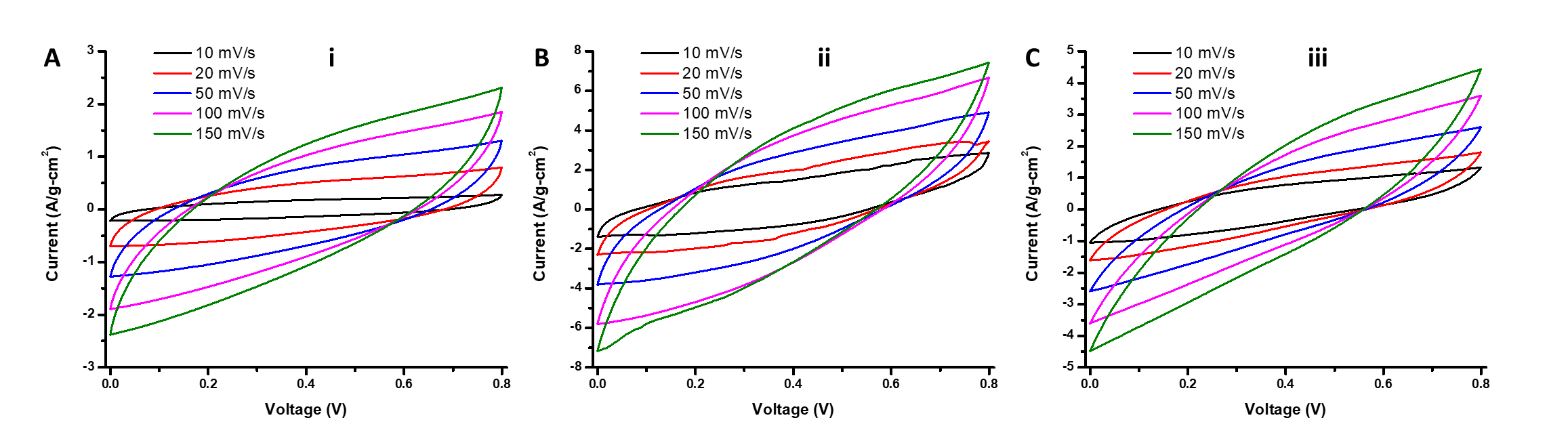


**Figure S7.** CV profiles of the system in a device for **A.** state **i**, **B.** state **ii**, and **C.** state **iii** at different scan rates.


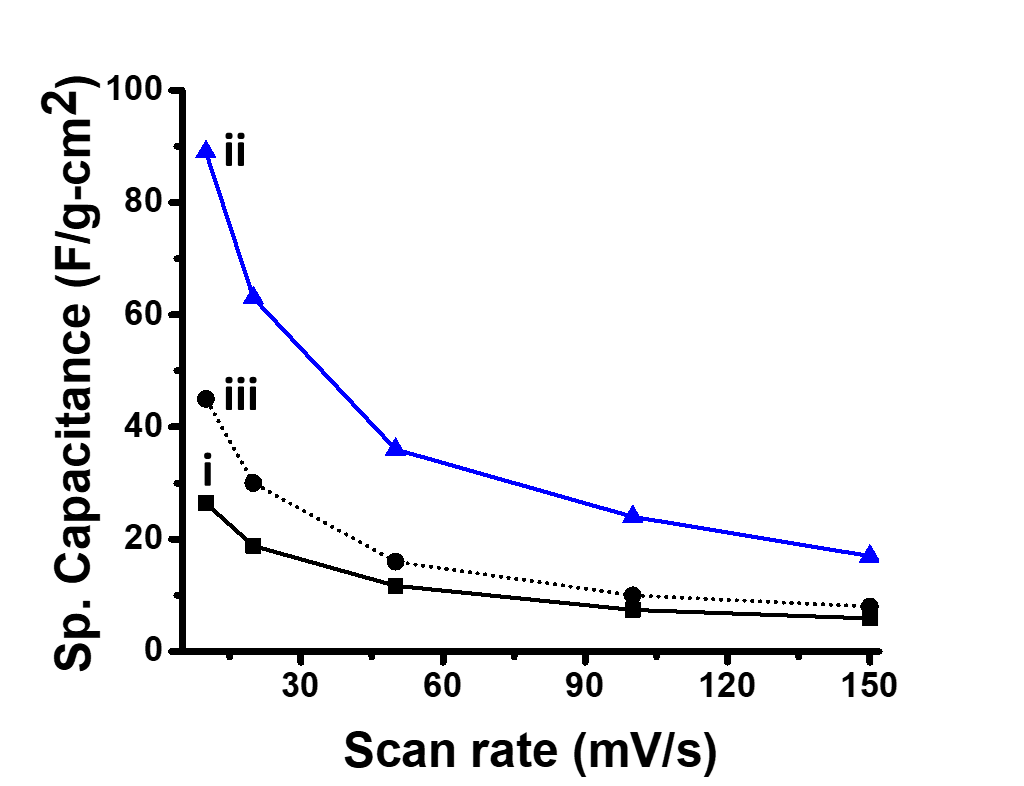


**Figure S8.** Specific capacitance vs scan rate plot for states **i**, **ii**, and **iii** in a device.

**Table S2.** Specific capacitance values at different scan rates for states **i**, **ii**, and **iii** in a device.

| Scan rate (mV/s) | Specific Capacitance for state i (Fg^-1^cm^-2^) | Specific Capacitance for state ii (Fg^-1^cm^-2^) | Specific Capacitance for state iii (Fg^-1^cm^-2^) |
| --- | --- | --- | --- |
| 10 | 26 | 92 | 45 |
| 20 | 19 | 63 | 30 |
| 50 | 12 | 36 | 16 |
| 100 | 7.4 | 24 | 10 |
| 150 | 6 | 17 | 8 |


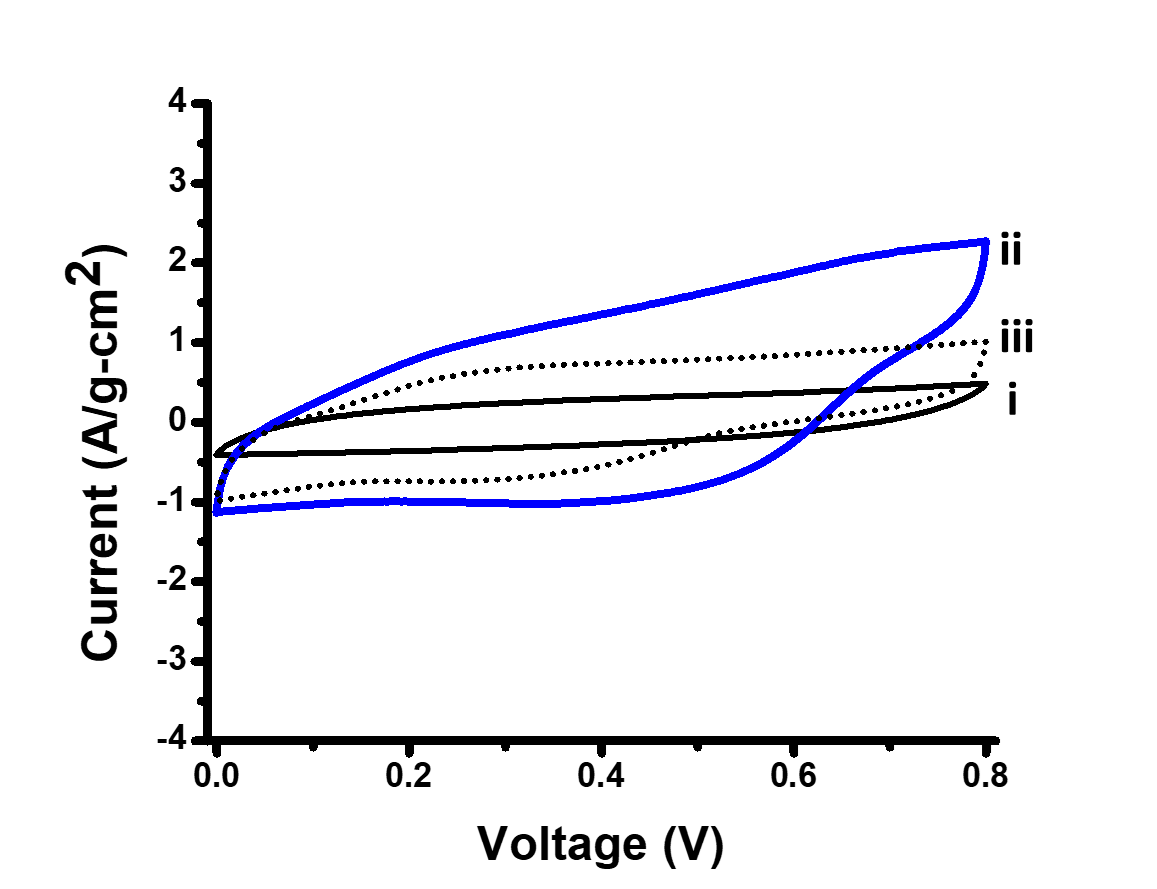


**Figure S9.** CV profile of the device for states **i**, **ii**, **iii** at 10 mV/s scan rate using a quartz cuvette.

**Figure S10.** Effect of heat on the CV profile of the device (state **iii**) at a scan rate of 10 mV/s.


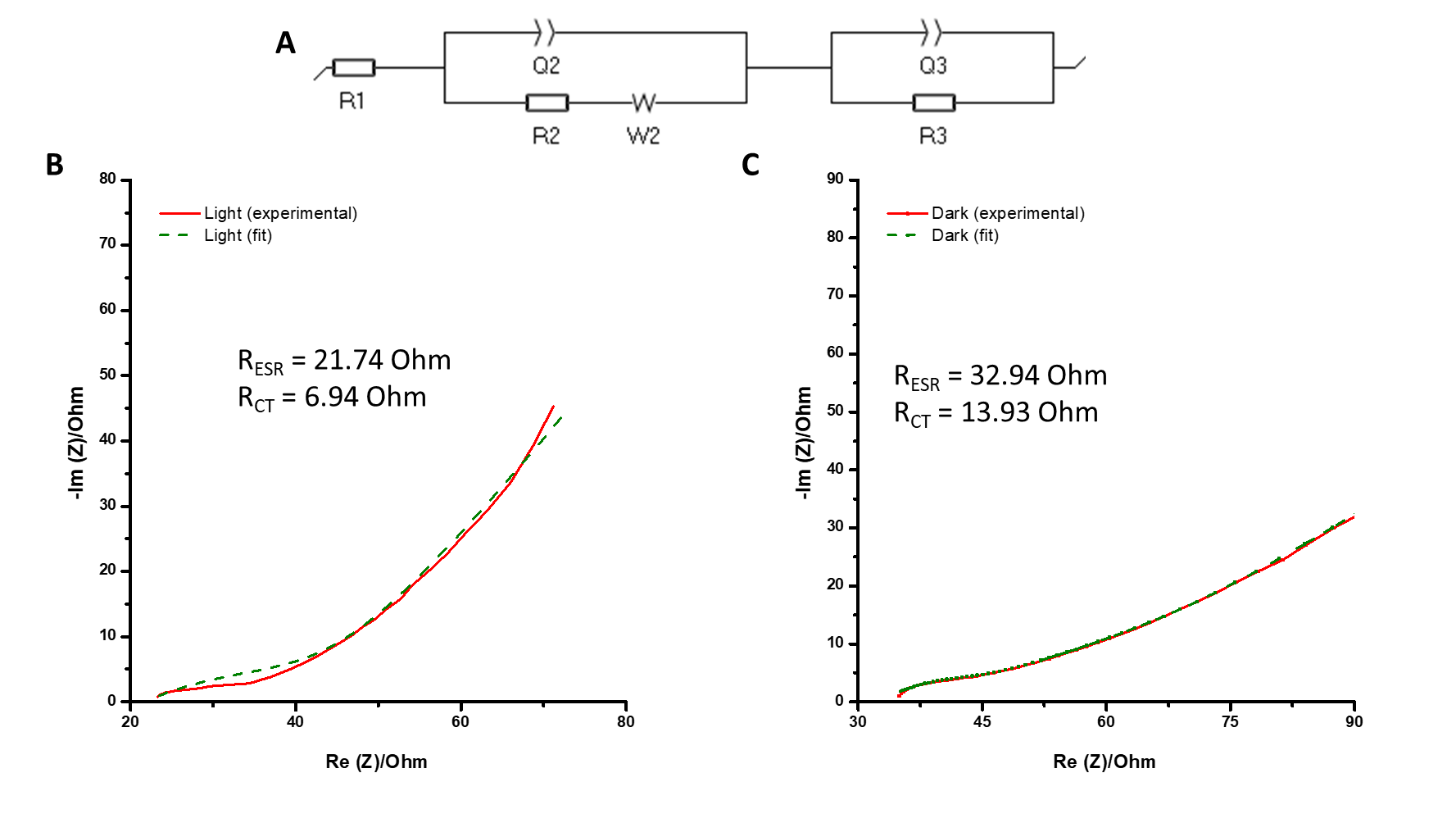


**Figure S11.** **A.** Electrical circuit for a non-ideal capacitor. Experimental and fitted EIS spectra for **B.** state **ii**, and **C.** state **iii** in a device.


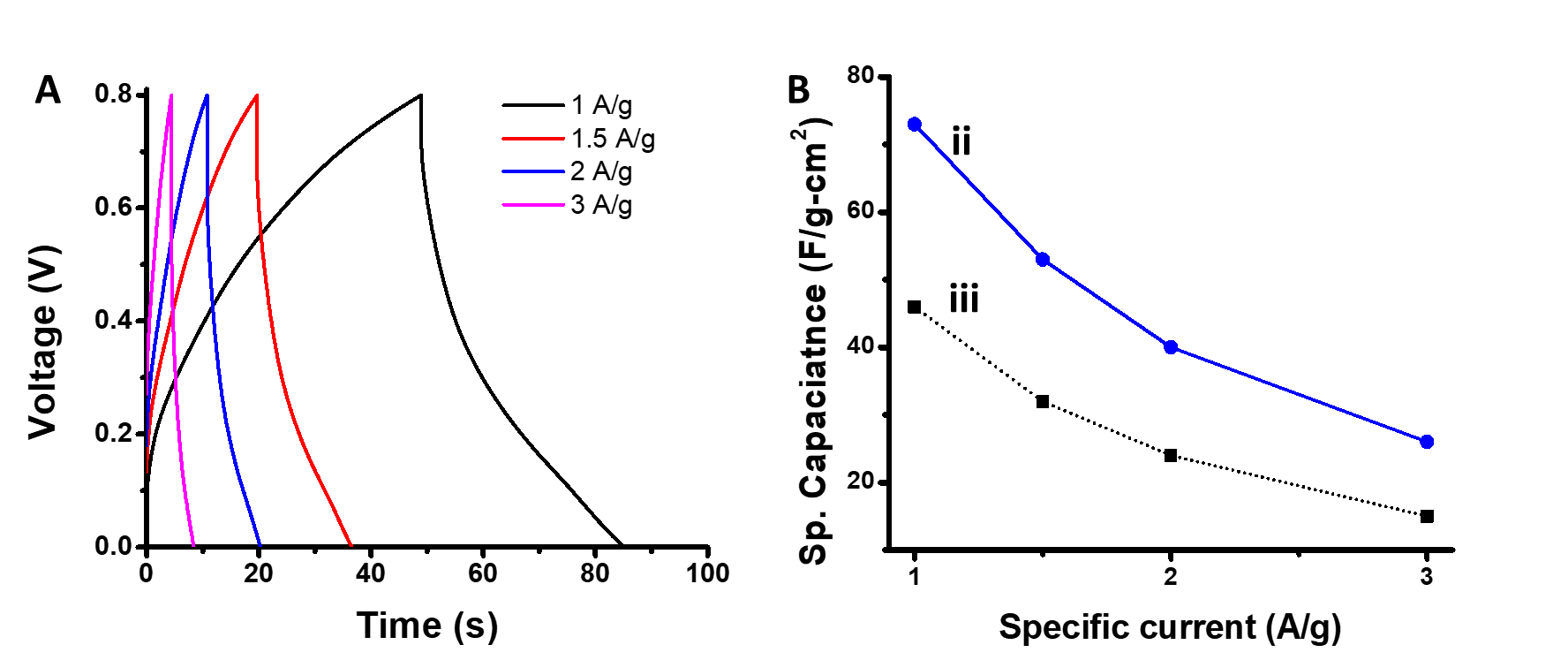


**Figure S12.** **A.** GCD profiles of the system in a device under dark (state **ii**) at different current densities. **B.** Specific capacitance vs current density curves obtained from GCD under light (state **ii**) and dark (state **iii**) conditions in a device.

**Figure S13.** Self-discharge profile of the device charged at 1 A/g specific current under light.

**Table S3.** Discharge times and specific capacitance values obtained for a device from GCD under light (state **ii**) and dark (state **iii**) conditions at different current densities.

| Current density (A/g) | Discharge time (s) at state ii | Specific Capacitance (Fg^-1^cm^-2^) at state ii | Discharge time (s) at state iii | Specific Capacitance (Fg^-1^cm^-2^) at state iii |
| --- | --- | --- | --- | --- |
| 1 | 60 | 73 | 37 | 46 |
| 1.5 | 28 | 53 | 17 | 32 |
| 2 | 16 | 40 | 9.5 | 24 |
| 3 | 7 | 26 | 4 | 15 |

References

[1] S. Biswas, N. Shauloff, R. Bisht, R. Jelinek, *Adv. Sustain. Syst.* **2023**, *7*, 2300035.
